# A scoping review on information and support needs regarding cancer and work: part I – cancer survivors

**DOI:** 10.1007/s11764-026-01994-7

**Published:** 2026-03-04

**Authors:** J. M. Mollet, I. L. Bakker, P. M. V. Janse, S. F. A. Duijts, A. G. E. M. de Boer, F. van Ommen, P. Coenen, M. A. Greidanus, S. J. Tamminga

**Affiliations:** 1 Department of Public and Occupational Health, Amsterdam , UMC Location University of Amsterdam, Amsterdam, The Netherlands; 2 Amsterdam Public Health Research Institute, Societal Participation and Health, Amsterdam, The Netherlands; 3 Netherlands Comprehensive Cancer Organization (IKNL), Research and Development, Utrecht, Netherlands; 4 Department of Medical Psychology, Amsterdam , UMC Location Vrije Universiteit, Amsterdam, The Netherlands; 5 Cancer Center Amsterdam, Cancer Treatment and Quality of Life, Amsterdam, The Netherlands; 6 Department of Public and Occupational Health, Amsterdam , UMC Location Vrije Universiteit, Amsterdam, The Netherlands

**Keywords:** Cancer survivorship, Work, Scoping review, Information needs, Support needs

## Abstract

**Purpose:**

A better understanding of survivors’ information and support needs could guide the design of more meaningful and effective interventions. This scoping review examines cancer survivors’ needs in the context of cancer and work.

**Methods:**

For this scoping review, we searched 5 databases for studies published in 2014–2024 reporting on cancer survivors’ information and support needs related to cancer and work. Data were extracted and identified needs were qualitatively synthesized using the Arena model of work disability.

**Results:**

Eighty-five studies from 22 countries were included, reporting on 26 themes concerning information needs and 56 themes concerning support needs. Within the personal system, survivors need information on how cancer and treatment affect work, and they need support through understanding from employers and colleagues. Within the workplace system, needs included information on return to work timing and phases, and support via flexible, personalized work adjustments. Within the healthcare system, survivors need information on cancer’s impact on work (dis)ability, and support includes tailored advice and encouragement. Survivors emphasized the importance of work being proactively addressed in clinical care. Within the legislative and insurance systems, needs related to information on employee rights and support in navigating disability benefit procedures were identified.

**Conclusions:**

The diversity in needs suggests that interventions should be tailored to individual cancer survivors, while considering broader contextual factors Future interventions should include multiple stakeholders, since needs exist throughout the systems of the Arena model.

**Implications for Cancer Survivors:**

There is a need for tailored interventions to support work participation of cancer survivors.

## Introduction

There is a growing recognition for the importance of work for cancer survivors [1] and there is a broad consensus that, for many cancer survivors, work offers more than just financial security. It provides daily structure, social connection, and a sense of purpose [1, 2], and for cancer survivors, these additional benefits could play a vital role in recovery and overall quality of life [3]. Internationally, there is a shift from focusing solely on *how long* people live after a cancer diagnosis to also considering *how well* they live[4]. This focus is particularly relevant, as recent advancements in cancer treatment—including less invasive and more targeted procedures—have made staying at work during treatment and/or returning to work afterwards a realistic, relevant, and meaningful goal for a growing number of survivors [5–7]. This is increasingly acknowledged in various national and international cancer plans. For example, in Europe’s Beating Cancer Plan, stakeholders recognize work reintegration as an important topic within its broader objective of improving the quality of life of cancer survivors [4]. Moreover, in the Netherlands Cancer Agenda, sustainable work participation of cancer survivors has explicitly been identified as one of its key goals for the coming years [8].

Over the past decades, numerous interventions have been developed to improve the work participation of cancer survivors, by supporting return to work and/or work maintenance [9]. Despite the rigorous design of many interventions [10–13], effectiveness studies provide limited evidence that these interventions successfully support work participation [9]. Given the limited effectiveness of existing interventions [9] and the growing recognition of the role of work in cancer survivorship [1, 8], there is a clear need for more evidence to guide the development of more effective interventions. A crucial first step in this process is to systematically scope the information and support needs experienced by cancer survivors in relation to staying at or returning to work. Mapping out these needs requires a well-structured framework, such as the 'Arena in work disability prevention model' (hereafter: Arena model) [14, 15] that captures the broad context in which these needs may be experienced. This model distinguishes several systems: the *personal system*, the *workplace system*, the *healthcare system*, and the *legislative and insurance systems,* and has been used multiple times to organize various factors that affect work participation of cancer survivors and those with other illnesses [16, 17].

Therefore, the aim of this scoping review was to examine cancer survivors’ information and support needs related to cancer and work. In this study, the term ‘cancer survivor’ refers to an individual from the moment of cancer diagnosis onward. This definition is consistent with the widely used definition of ‘cancer survivorship’ as a process that begins at diagnosis and extends across the lifespan [18]. A better understanding of these information and support needs can inform the development of future, multi-component interventions that are more closely aligned with what cancer survivors actually require. Embedding these insights within the personal, workplace, healthcare, and legislative and insurance systems will facilitate context-sensitive strategies that are meaningful and effective in improving survivors’ work participation.

## Methods

### Design

For this scoping review, a search was conducted for a broader research question than presented in the current article, i.e., not only including the needs of cancer survivors (part I), but also of employers and other stakeholders (part II). In this article, we report on the results of cancer survivors only. The checklist Preferred Reporting Items for Systematic reviews and Meta-analyses extension for Scoping Reviews (PRISMA-ScR) was used as a framework for this review [19]. The protocol for this review was not registered.

### Search strategy

The literature search was conducted by an experienced clinical librarian and checked by two authors (ST and JM). The following databases were searched: Medline, Embase, PsycINFO, Cinahl, and Scopus. Medical subject terms (MeSH terms) and title and abstract words (TIAB) related to ‘cancer’, ‘employment’, ‘worker’, ‘employer’, ‘professional’, ‘support’, ‘information’, and ‘needs’ were combined and adapted for the different databases. Randomized controlled trials were excluded by using the ‘NOT’ operator. An overview of the search strings can be found in Appendix A. Filters were applied to limit records to those published from January 2014 up to and including August 2024, written in English or Dutch, and available in full text. The publication timespan starting from 2014 was chosen because of advancements in cancer treatment, which has improved survival rates. This also has led to evolving implications in the field of cancer and work [5].

### Article selection

Articles were eligible for inclusion if: (1) the study design was quantitative (e.g., surveys), qualitative (e.g., interviews, focus groups) or mixed-method (including e.g., process evaluations or descriptions of intervention development); (2) they were original, peer-reviewed research articles; (3) the full text was available in English or Dutch; (4) the study sample consisted of cancer survivors; and (5) the results section described at least one cancer survivor’s information or support need regarding cancer and work. Studies were excluded if the design concerned an effectiveness study (such as a randomized controlled trial), as emerging needs are rarely reported in such studies.

The article selection process consisted of two steps. First, title and abstract screening was performed by two authors (ST and JM) simultaneously, using the oracle mode in ASReview. We pre-trained the model (default setup: TF-IDF; Naïve Bayes; Maximum; Dynamic resampling) by uploading five relevant [20–24] and five irrelevant research articles as prior knowledge. Based on previous research in which machine learning was used to support the screening process, we used a stopping criterion of 50 consecutive irrelevant articles, assuming that all subsequent articles were likely to be irrelevant and could be excluded [25]. Second, a double-blind full text screening was conducted by combinations of two authors (ST, MG, AdB, SD, and JM) using Covidence [26]. In case of disagreement during the article selection, articles were discussed among two authors (ST and JM) until consensus was reached.

### Data extraction

The data extraction was divided among five authors (ST, MG, AdB, SD, and JM) and it was not double-checked. However, in case of doubt during data extraction, discussion between two researchers (ST and JM) were held until consensus was reached. We used four predesigned tables: 1) study characteristics (e.g., author, country, study design), 2) sample characteristics (e.g., cancer type, occupation), 3) information needs, and 4) support needs (supplementary file A). In the current scoping review, survivors’ information and support needs in the context of cancer and work were explored. The definition of a support and information need is derived from patient care, i.e. *a support need refers to any form of assistance required, while an information need refers to the relevant knowledge or resources necessary to enhance a patient’s quality of life, understand and manage their condition, or facilitate recovery.* These needs can be diverse and multifaceted, encompassing various aspects of a patient’s (work) life and healthcare journey. The concept of support and information needs is integral to delivering holistic, person-centered care, ensuring that patients receive comprehensive support and information tailored to their individual circumstances. This revised definition of information and support needs draws upon the definition previously applied to the needs of intensive care survivors [27].

### Data synthesis

The Arena model was used for the categorization of information and support needs, as this model describes four main systems that could influence the work participation of cancer survivors: personal system, healthcare system, workplace system, and legislative and insurance systems [14, 28]. Within those main systems, themes were identified by one author (JM) and checked and supplemented by another (MG). For this data synthesis, we used the qualitative data analysis software MAXQDA [29].

## Results

### Study characteristics

**Fig. 1:**
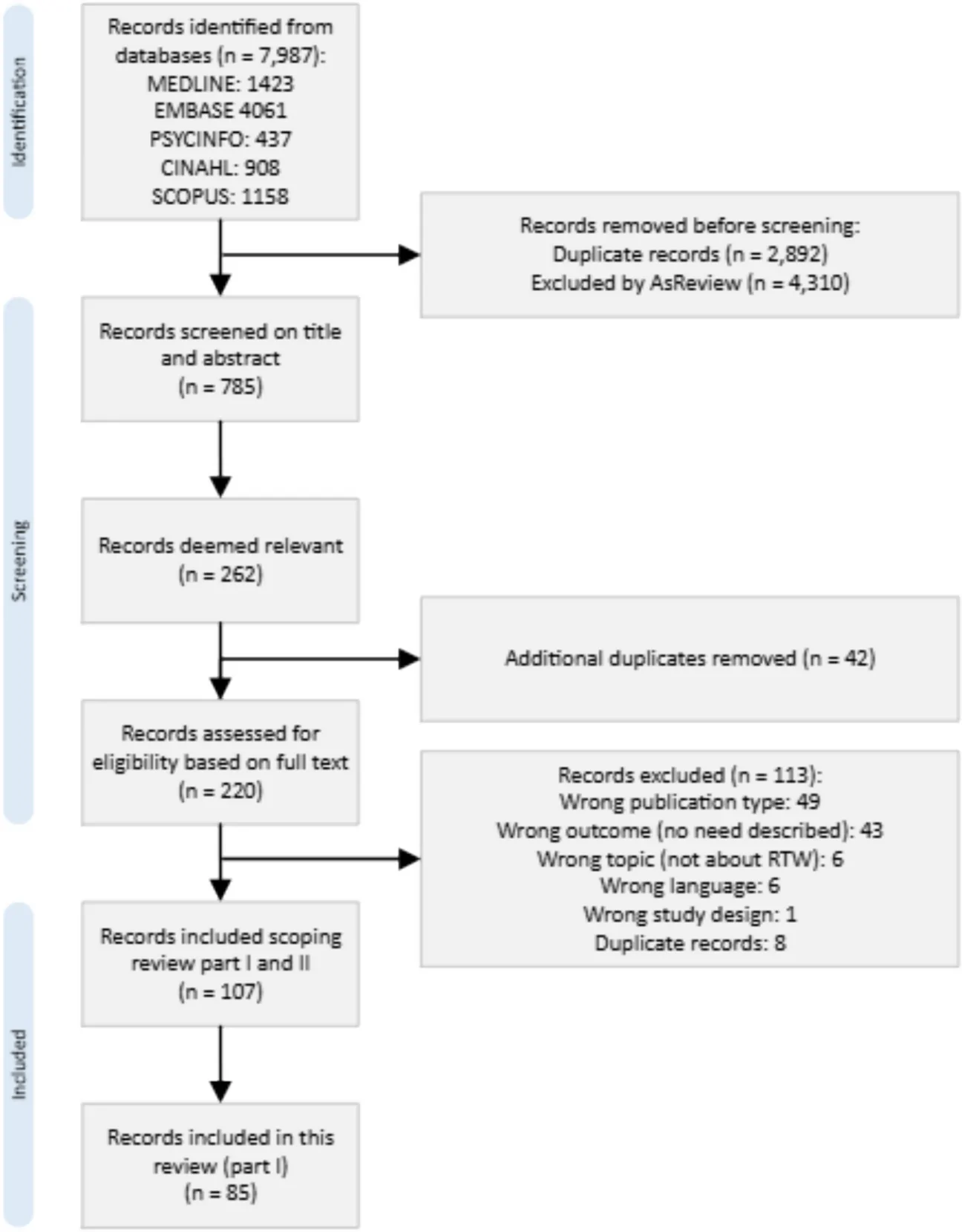
PRISMA flowchart of study selection and screening

Our search strategy initially identified 7,987 records, of which 2,892 were duplicates. Applying a stopping criterion of 50 consecutive irrelevant articles, we screened 785 titles and abstracts. Of these, 262 were deemed relevant. After removing an additional 42 duplicates, 220 full-text articles were assessed for eligibility. Next, 113 full-text articles were excluded, mostly due to a wrong publication type or wrong outcome—no need was expressed by the stakeholder in question -, resulting in a final selection of 107 articles. It is correct that at various stages duplicates were identified; this is because the same record from different databases is unfortunately not always automatically recognized as duplicate. For the current paper, 85 articles on the information and support needs of cancer survivors were included (Fig. 1). The other 22 studies identified information and support needs of employers and other stakeholders and thus fell outside the scope of this article and were described in part II.

**Fig. 2:**
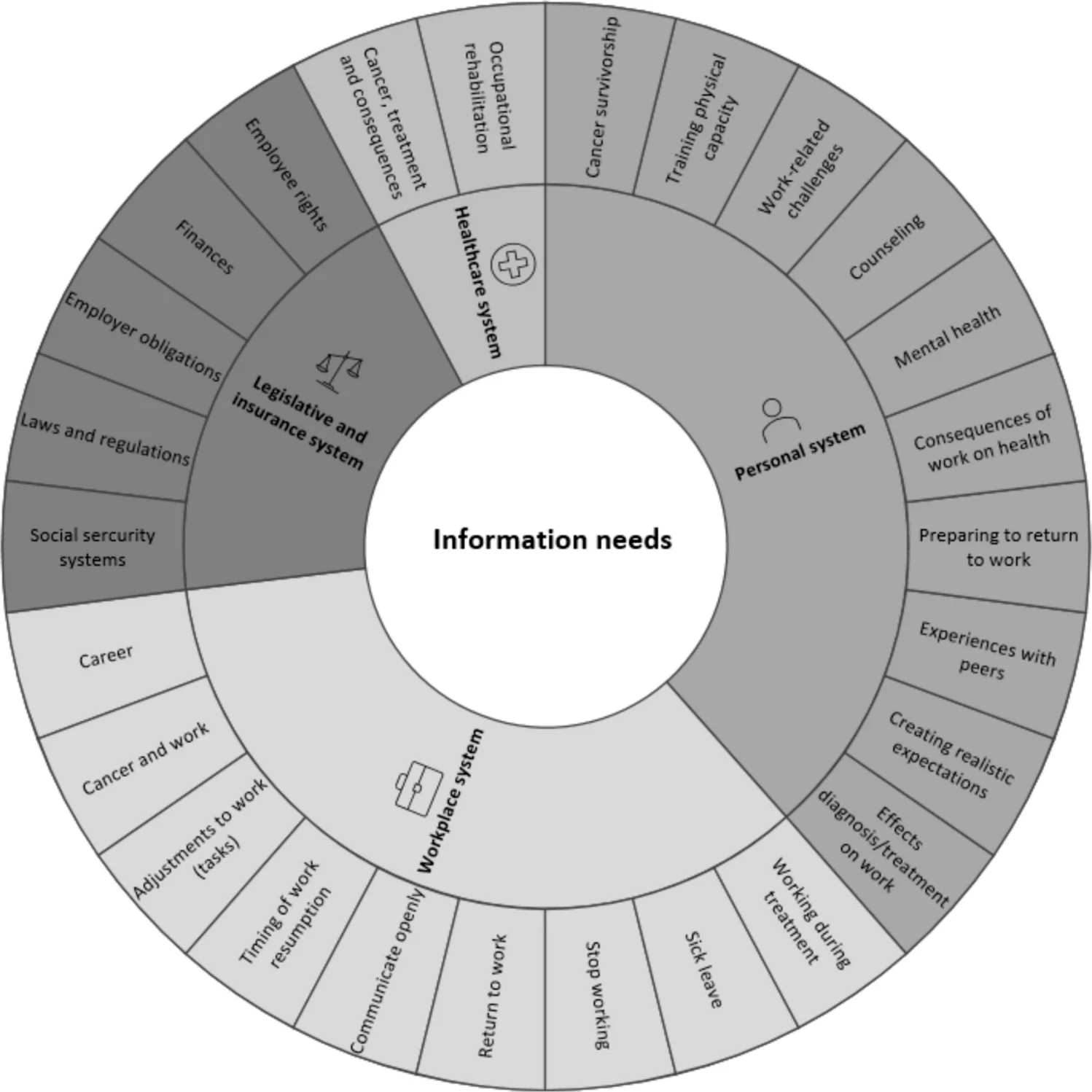
Identified themes of information needs corresponding to each of the four systems of the Arena model

**Fig. 3:**
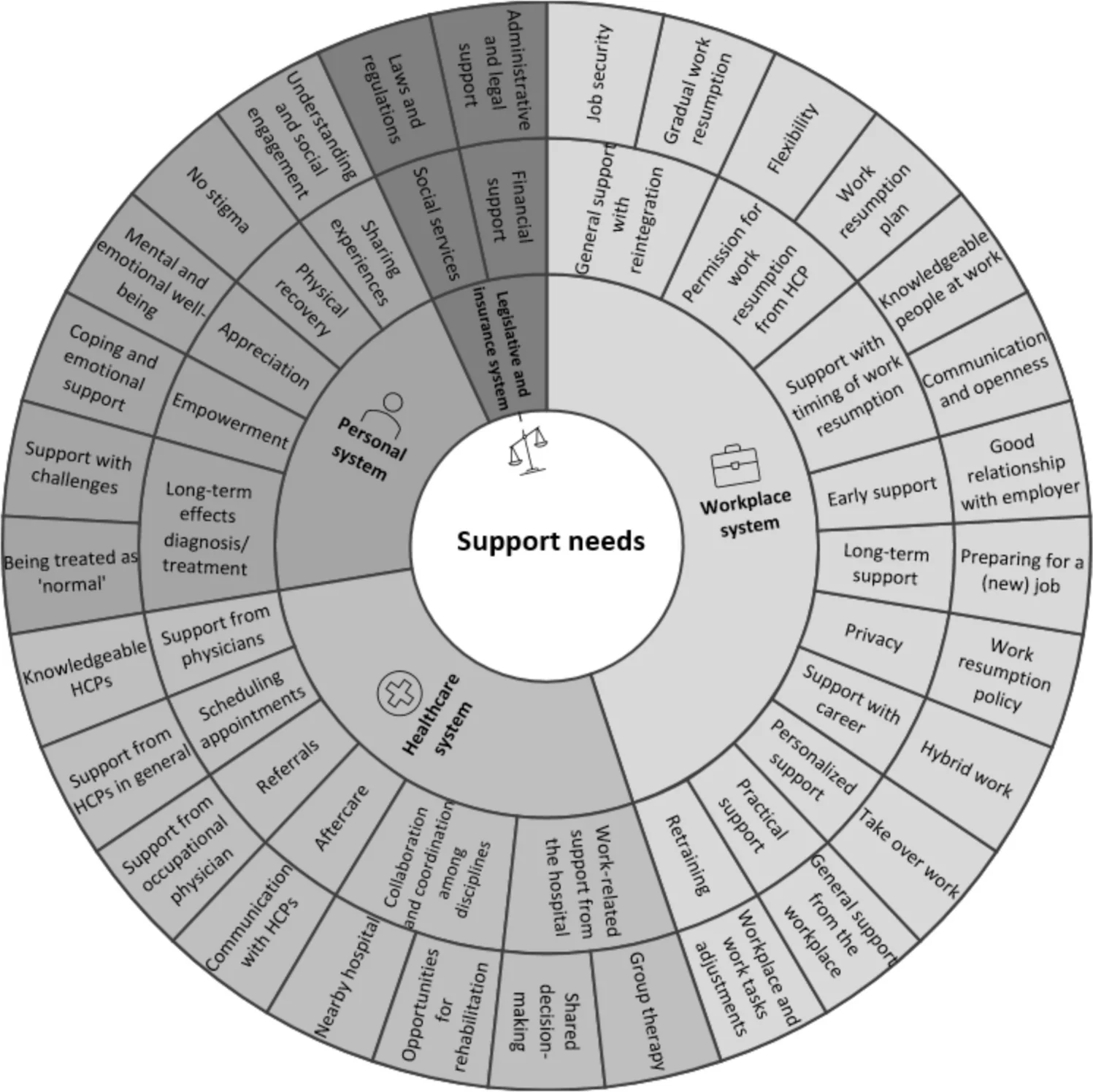
Identified themes of support needs corresponding to each of the four systems of the Arena model. Note: The arrangement of themes does not imply any hierarchy or relative importance. The arrangement was a practical design choice to clearly present all identified themes in one figure. Abbreviation: HCP = Healthcare professional

A total of 22 countries were represented in the included studies, with most research conducted in the Netherlands (N = 15, 17,6%), United States (N = 14, 16,5%), Canada (N = 14, 16,5%), and Australia (N = 9, 10,6%). Nine studies were longitudinal studies; 76 studies were cross-sectional. The included studies covered a wide variety of cancer types (e.g., breast cancer, prostate cancer, and colon cancer) and a broad variety of occupations among cancer survivors. An overview of the study characteristics is provided in Table 1. An overview of the synthesized needs in themes is provided in Figs. 2 and 3 respectively. The data extraction per included articles is provided in supplementary file B.

**Table 1:** Study and patient characteristics

		*N* (%)
Country	Netherlands	15 (18%)
	United States	14 (17%)
	Canada	14 (17%)
	Australia	9 (11%)
	United Kingdom	6 (7%)
	China	3 (4%)
	Iran	2 (2%)
	Korea	2 (2%)
	Norway	2 (2%)
	Spain	2 (2%)
	South Africa	2 (2%)
	Denmark	2 (2%)
	France	2 (2%)
	Other	9 (11%)
Study design	Cross-sectional	76 (89%)
	Longitudinal	9 (11%)
Data collection method	Individual interview	67 (79%)
	Questionnaire	18 (21%)
	Focus group	14 (17%)
Cancer diagnosis is	Breast cancer	25 (29%)
	Brain cancer	4 (5%)
	Colorectal cancer	2 (2%)
	Head and neck cancer	3 (4%)
	Prostate cancer	2 (2%)
	Multiple myeloma	2 (2%)
	Gastrointestinal cancer	1 (1%)
	Hematologic cancer	1 (1%)
	Ovarian cancer	1 (1%)
	Thyroid cancer	1 (1%)
	Various	40 (47%)
	Not reported	3 (4%)

Note: The total numbers for Study Design and Methodology may exceed the number of included studies, as individual studies may have employed multiple designs or methodologies

### Information and support needs

#### Personal system

Cancer survivors were found to experience a need for information on cancer survivorship [30], effects of diagnosis/treatment on work [24, 30–41], experiences of peers [22, 39, 42], mental health [42, 43], counseling [24] and training physical capacity [44], and on the consequences of work on health [45, 46]. Additionally, they were found to experience a need for support to deal with the (long-term) effects of their diagnosis and/or treatment on work [6, 22, 23, 31, 33, 35, 36, 41, 47–55], including support with mental and emotional well-being [23, 31, 34, 45, 56–61], physical recovery [61–64] and the need for support by means of sharing experiences with peers [23, 58, 65].

Cancer survivors reported a support need for understanding and social engagement from stakeholders tailored to their personal situation [6, 21–23, 32, 33, 35, 36, 45, 47–49, 52, 54–56, 58, 61, 66–74], including coping and emotional support [21, 40, 50, 57, 66, 67, 75, 76], expressions of appreciation [35, 50, 54] and the desire of being treated as ‘normal’ [36, 38, 50, 54, 77], with no stigma [58, 68]. Moreover, cancer survivors experienced a need for support from the workplace through empowerment, enabling them to contribute meaningfully to their work [23, 66].

Regarding work readiness, survivors experienced a need for information for (creating) realistic expectations [32, 35, 43, 61], for preparing to return to work [44, 53, 61] and for dealing with work-related challenges [33, 78] as well as support with challenges they experience when returning to or maintaining work. This support could be provided through discussions with healthcare professionals, people at work, or loved ones [39, 40, 59, 63, 64, 78, 79].

#### Workplace system

Related to the workplace system, information needs regarding cancer and work [22, 48, 57, 78, 80] were found including information on adjustments to work (tasks) [45, 46, 57], on the right timing of work resumption [45, 81, 82], career prospects [44, 56, 75, 79] and on how to communicate openly [24, 57, 67]. Moreover, cancer survivors reported information needs related to specific phases of the return to work journey, i.e.: working during treatment [34], sick leave [24], planning to and actual return to work [23, 51, 66], and stop working [24].

Support needs referred to the whole return to work journey including early support [83, 84] and long-term support [71, 85]. The type of support varied from general support from the workplace [6, 49, 67, 75, 81, 82, 86, 87] and general support with reintegration [35, 46, 56] to more specific needs. That is, support with workplace and work tasks adjustments [6, 21, 22, 30, 32, 33, 35, 36, 45, 48–52, 57–59, 64, 67–69, 71, 73, 77, 86–95], hybrid work [35, 64, 67], practical support [35, 36, 50, 55, 59, 66, 89, 94], colleagues that (partially) take over work from them [40, 54, 58, 80], flexibility [6, 23, 24, 47, 50, 51, 53–55, 58, 59, 64, 67, 72, 77, 80, 91, 92, 94–96], job security [38, 51, 54, 92], work resumption policy [94], possible career prospects [35, 56, 79, 88, 97], e.g. support from an employer or personal coach in preparing for a (new) job [61, 88] or exploring retraining possibilities [21, 56, 87–89], and support with formulating a work resumption plan [21, 33, 48, 50, 64, 96], including timing of work resumption [57, 72], gradual work resumption [59, 77, 91, 93] and permission for work resumption from healthcare professionals [6].

Cancer survivors were also found to need support in terms of a good relationship with their employer [69] and colleagues, by means of continuous, empathetic, sensitive, and effective communication and openness [6, 21–23, 31, 35, 36, 50, 57, 59, 63, 70, 77, 81, 87, 95, 96, 98], while respecting cancer survivor’s privacy [6, 58, 69]. This support should come from knowledgeable people at work, with an understanding of cancer and its impact on work and well-being [21, 22, 41, 49, 52, 54, 58, 62, 66, 90, 98], and it should be personalized support to meet the specific needs of the individual [7, 47, 50, 59, 73, 83].

#### Healthcare system

Related to the healthcare system, cancer survivors only reported needs for information regarding cancer, treatment, and its consequences [22, 35, 38, 40, 46, 57, 61, 62, 99, 100] and occupational rehabilitation [22, 23, 51, 101].

Cancer survivors reported a needing support from healthcare professionals in general [23, 38, 40, 55, 63, 73, 74, 102], with one study reporting a need for support through group therapy provided by clinicians [57]. Moreover, they expressed specific needs for support from physicians [6, 37, 40, 67, 72, 103], for example through encouragement [67], and support from occupational physicians [7, 22, 84, 98, 104].

In addition, a need for work-related support from the hospital was found [7, 21, 23, 72, 73, 98, 105] including support with organizing treatment in a nearby hospital [55], ensuring flexibility in scheduling appointments (i.e., taking the patient’s work into account) [55, 72], and referrals to other specialists [23, 31, 103]. Besides, support from other healthcare organizations such as receiving aftercare following cancer treatment [41, 57–59] and opportunities for rehabilitation services such as occupational therapy and physical therapy were found [63, 64, 106]. One study emphasized the importance of return to work as an integral part of survivorship care [59].

A need for high-quality communication between professionals and the patient [100] and shared decision making, for example regarding the timing of work resumption [30] was found, as well as optimal collaboration and coordination among disciplines, such as healthcare professionals, occupational physicians, and employers [21, 22, 88, 98, 103, 105]. It was found that all of the abovementioned healthcare professionals should be knowledgeable healthcare professionals [21, 22, 31, 55, 58, 61, 70], which for occupational and insurance physicians requires in particular expertise in cancer [31, 104].

#### Legislative and insurance systems

Five information needs were found, related to the legislative and insurance systems. That is, the need for information about: 1) finances, e.g. the financial impact of a cancer diagnosis [37, 45, 57, 63, 65, 87, 93, 99], 2) laws and regulations, particularly regarding sick leave [63, 105], 3) employer obligations [107], 4) employee rights [21, 24, 43, 59, 62, 63, 99, 101, 107] and 5) about how social security systems work [35].

In addition, four support needs were found, that is: 1) a need for financial support, including (extended) paid sick leave, paid time off during appointments, employer-covered health insurance, and financial support for education [6, 33, 45, 49, 50, 55, 56, 58, 60, 63, 64, 76, 79, 86, 89, 90, 91, 92], 2) support to navigate laws and regulations, for example those related to employee rights [6, 71], 3) support from social services, for example regarding negotiations with the workplace about return to work [59], and 4) administrative and legal support, including support with claiming disability benefits, completing leave related paperwork, and managing insurance matters [23, 33, 49, 56–58, 62, 64, 87, 92, 100].

## Discussion

### Main findings

Across all systems, cancer survivors reported a diverse range of information and support needs related to cancer and work with some needs reported by almost all included studies and some needs by only one study. Within the personal system, survivors need information on the effects of diagnosis and treatment on work, and on realistic expectations for returning to work. They needed support for managing long-term physical and emotional consequences, mental well-being, and for understanding from stakeholders tailored to their situation. In the workplace system, key information needs concerned work adjustments, timing and phases of work resumption, while support needs included flexible work arrangements, job security, reintegration assistance, and empathetic communication from employers. Within the healthcare system, survivors required information on treatment consequences and occupational rehabilitation, alongside support through collaboration between disciplines, emphasizing the role and importance of knowledgeable healthcare professionals. In the legislative and insurance systems, survivors needed clear information on finances, laws, rights, and social security, as well as support navigating regulations, accessing financial assistance, and managing administrative processes. These findings underscore the importance of tailoring interventions to address interconnected needs across personal, workplace, healthcare, and policy domains.

### Interpretation of findings

Both our research and previous studies examining other patient populations show that information and support needs of patients regarding work vary between individuals and consider a diversity of stakeholders [108, 109]. This finding emphasizes that a one-size fits all approach does not suffice and emphasizes the need for personalized holistic interventions [39].

Furthermore, the included studies originated from a variety of countries worldwide but were predominantly conducted in high-income countries, where, for example, structured occupational healthcare services are more commonly available [110, 111]. However, it is important to acknowledge that although studies conducted in high-income countries were dominant, substantial heterogeneity in context, return to work policies, and stakeholders involved existed between the included studies [112]. For example, the most frequently represented countries in this review were the Netherlands and the United States, and a comparison showed substantial differences in national legislation and organizational structures. While the Dutch system is characterized by legally mandated sick leave and structured return to work follow-up regimes [113], the United States relies largely on employer-based arrangements with limited statutory protection [114]. Moreover, the occupational health service coverage [110] and access to occupational physicians [111] are more commonly available in the Netherlands than in the United States. Therefore, we found that the identified needs are shaped by the local context in three ways. First, the extent to which information and support needs are already met, shape whether identified needs are more ‘general’ or more ‘specific’. To give an example, in the study of Lee et al., the authors reported one general Cancer and Work information need, i.e. ‘the need for information and resources on how to prepare well for work’ [53], compared to, for example, the study from Olischlager et al. that reported several Cancer and Work information needs, such as: ‘the need for a guideline depicting important medical and occupational information’ [22]. Second, legislation on sick-leave and return to work affects stakeholders’ role and responsibility, which in turn shape information and support needs of cancer survivors. This entails, for example, the need to receive support from an occupational physician, which is in line with studies from the Netherlands [7, 22, 84, 104] and France [98], while absent in many other studies. Third, the type of legislation on sick-leave and return to work (if any) varies between countries [115]. This determines which information is relevant as well as what kind of support cancer survivors require. For example, employer-based health insurance applies mostly to cancer survivors in the United States and Canada and is therefore reported as an information need in studies from these countries [92], while in a study from Iran the authors reported on the need for more financial security [45]. The finding that local context shape needs, means that interventions should not only be personalized and holistic but also adapted to local contexts.

### Strengths and limitations

The strength of this scoping review is its comprehensive data extraction and data synthesis, which contributed to a transparent and extensive overview of reported information and support needs of cancer survivors. Several limitations should also be noted. It appeared that it was sometimes difficult to disentangle from a qualitative study whether the authors reported on a ‘need’ or an ‘experience’. This is important since these are not necessarily the same. For that reason, in the case of doubt, we discussed findings within the project team until consensus was reached. We furthermore reported the raw data as extracted from the included studies in the supplementary file for transparency. Comparable, it appeared that it was sometimes difficult to disentangle whether the authors report on an ‘information’ or a ‘support’ need. We therefore defined both types of needs and again reported the raw data in the supplementary file. As many of this review’s authors are experts on cancer and work, reflexivity bias can be an issue. This form of bias refers to the subjective influence of the researchers’ beliefs, experiences, and preconceptions on the research process [116]. We have tried to minimize this form of bias by including all authors in interpreting the results in multiple phases of the project.

### Implication for future research and clinical practice

Considering the amount, diversity and complexity of the identified needs in all systems of the Arena model of work disability, we recommend first and foremost developing tailored holistic interventions. Part of this is studying by whom, when and how a need should be met, also given the local context (e.g. disability regulations, division of tasks between professionals). Moreover, future research should focus on strengthening the study’s findings through subgroup analyses and contextual comparisons, stratified by institutional context (e.g. employer liability and sick leave policies), cancer characteristics (e.g. type and stage), occupation type, and treatment phase. Furthermore, incorporating quality of evidence and prioritization of needs (e.g. importance and intervenability) should be added to better inform future intervention development. Our finding that needs are present in all systems of the Arena model, combined with the identified stakeholder needs (part II), also advocates for developing interventions that do not target one stakeholder (e.g. healthcare professional, employer) only, but offers a system approach including enhancing communication and collaboration between stakeholders acting in different systems. We considered cancer survivors’ perspectives only. We did not validate their needs with theoretical reasoning nor with stakeholder knowledge and experiences. Addressing specific information or support needs does not automatically mean that it enhances the sustainable work participation of a cancer survivor. For instance, some types of support could actually delay return to work. This is for example seen, in a study in which sick-listed workers with common mental disorders were gradually exposed to more demanding work situations [112]. For practice, this means that it must be determined for every individual what appropriate support entails.

As supporting work participation of cancer survivors takes place at the intersection of the personal, workplace, healthcare, legislative and insurance systems, we advocate for: 1) healthcare workers who actively ask about the work situation of their cancer patients, 2) occupational health and safety professionals, HR-professionals, employers, supervisors and colleagues who familiarize themselves with what it could mean to be diagnosed with cancer and listen to what the individual worker diagnosed with cancer need, and 3) work on cross-system care.

## Conclusion

The number and diversity in the nature and complexity of the identified needs indicate that interventions to support returning to and retaining work need to be personalized to the individual cancer survivor, to countries’ legislative system and the organization of clinical and occupational healthcare. Future interventions should include multiple stakeholders, since needs exist throughout the systems of the Arena model.

## Data Availability

The datasets generated during and/or analysed during the current study are available in the supplementary files.
